# Multiple proteins arising from a single gene: The role of the Spa33 variants in *Shigella* T3SS regulation

**DOI:** 10.1002/mbo3.932

**Published:** 2019-09-13

**Authors:** Mahendar Kadari, Dalila Lakhloufi, Valérie Delforge, Virginie Imbault, David Communi, Pierre Smeesters, Anne Botteaux

**Affiliations:** ^1^ Laboratory of Molecular Bacteriology Faculty of Medicine Free University of Brussels Brussels Belgium; ^2^ Mass Spectrometry and Proteomics Facility IRIBHM Faculty of Medicine Free University of Brussels Brussels Belgium; ^3^ Department of Pediatrics Academic Children Hospital Queen Fabiola Université libre de Bruxelles Brussels Belgium; ^4^ Tropical disease Group Murdoch Children’s, Research Institute Melbourne Vic. Australia; ^5^ Center for International Child Health University of Melbourne Melbourne Vic. Australia

**Keywords:** bacterial virulence, pathogenesis, secretion regulation, *Shigella flexneri*, type 3 secretion system

## Abstract

*Shigella* invasion and dissemination in intestinal epithelial cells relies on a type 3 secretion system (T3SS), which mediates translocation of virulence proteins into host cells. T3SSs are composed of three major parts: an extracellular needle, a basal body, and a cytoplasmic complex. Three categories of proteins are hierarchically secreted: (a) the needle components, (b) the translocator proteins which form a pore (translocon) inside the host cell membrane and (c) the effectors interfering with the host cell signaling pathways. In the absence of host cell contact, the T3SS is maintained in an “off” state by the presence of a tip complex. Secretion is activated by host cell contact which allows the release of a gatekeeper protein called MxiC. In this work, we have investigated the role of Spa33, a component of the cytoplasmic complex, in the regulation of secretion. The *spa33* gene encodes a 33‐kDa protein and a smaller fragment of 12 kDa (Spa33^C^) which are both essential components of the cytoplasmic complex. We have shown that the *spa33* gene gives rise to 5 fragments of various sizes. Among them, three are necessary for T3SS. Interestingly, we have shown that Spa33 is implicated in the regulation of secretion. Indeed, the mutation of a single residue in Spa33 induces an effector mutant phenotype, in which MxiC is sequestered. Moreover, we have shown a direct interaction between Spa33 and MxiC.

## Graphical abstract

We have shown that the Shigella spa33 gene, encoding a type 3 secretion system component, gives rise to 5 fragments of various sizes. Among them, three are necessary for type 3 secretion. For the first time, we have shown that Spa33 is implicated in the regulation of secretion and interacts with the gatekeeper protein, MxiC.

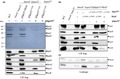

## 1 INTRODUCTION

Shigellosis was the second leading cause of diarrheal mortality in 2016, accounting for more than 200,000 deaths worldwide (Khalil et al., 2018). Symptoms of shigellosis are mainly due to the invasion of the colon associated with a severe inflammatory reaction and mucosal destruction (Sansonetti, Tran Van Nhieu, & Egile, 1999). The entry of *Shigella* into the host cell is mediated by the highly conserved type 3 secretion system (T3SS). T3SS spans the whole cell envelope translocating virulence proteins directly into the cytoplasm of the host cells (Cornelis, 2006; Galán & Wolf‐Watz, 2006), required for bacterial invasion, intracellular spread, and inhibition of the host immune defenses (Sansonetti, 2006; Schroeder & Hilbi, 2008). The T3SS is divided structurally into three parts: an extracellular needle, a transmembrane basal body, and a cytoplasmic bulb (Blocker et al., 2001; Burkinshaw & Strynadka, 2014; Chatterjee, Chaudhury, McShan, Kaur, & Guzman, 2013). At 37°C, the assembly of the basal body is triggered and the needle subunit MxiH is secreted through the T3SS together with the inner‐rod component MxiI (Magdalena et al., 2002). MxiH is a ~9 kDa conserved protein, which forms a ~50 nm long needle structure by polymerization (Blocker et al., 2001; Cordes et al., 2003; Fujii et al., 2012). Secreted proteins are divided into three classes: translocators, early effectors, and late effectors. In the absence of host cell contact, the translocators IpaD and IpaB maintain the T3SS in an inactive form by forming the needle tip complex (TC) (Blocker et al., 1999; Roehrich, Martinez‐Argudo, Johnson, Blocker, & Veenendaal, 2010). When T3SS is inactive, early effectors are presynthesized and stored within the bacterial cytoplasm with their cognate chaperones (Schroeder & Hilbi, 2008). Effector secretion is also prevented by a cytoplasmic gatekeeper protein, called MxiC (Botteaux, Sory, Biskri, Parsot, & Allaoui, 2009; Cherradi et al., 2013; Martinez‐Argudo & Blocker, 2010). Before secretion, transcription of late effectors is in an off‐state due to the presence of an anti‐activator, OspD1, binding to the transcriptional activator, MxiE. Moreover, the MxiE co‐activator, IpgC, is bound as a chaperone to IpaB and IpaC (Le Gall et al., 2005; Mavris, Sansonetti, & Parsot, 2002; Parsot et al., 2005).

Upon activation of T3SS by host cell contact, the translocators IpaB and IpaC, are inserted into the host cell membrane forming a translocation pore (Blocker et al., 1999; Veenendaal et al., 2007), and releasing IpgC in the cytoplasm. Pore insertion triggers a signal, probably transmitted through the needle, to allow MxiC and subsequent effector release, including OspD1 (Kenjale et al., 2005; Veenendaal et al., 2007). IpgC and MxiE together can activate x late effector transcription and subsequent secretion. Point mutations in two needle components (MxiH and MxiI) lead to an “effector mutant” phenotype defined by an absence of effector secretion while the translocators remains normally secreted (Cherradi et al., 2013; El Hajjami et al., 2018; Kenjale et al., 2005). Interestingly, deletion of *mxiC* in these strains restores their ability to secrete effectors (Cherradi et al., 2013; El Hajjami et al., 2018; Martinez‐Argudo & Blocker, 2010), suggesting that MxiC is involved in the regulation of effector secretion. A direct interaction between MxiC and MxiI has been shown in this process but no cytoplasmic component receiving the activation signal has been identified to date.

In *Shigella*, the cytoplasmic complex is composed of Spa33, Spa47, MxiK, and MxiN, forming a high molecular weight complex and serves as a sorting platform for T3SS substrates Spa33 is located beneath the basal body and interacts with the cytoplasmic moiety of the basal body proteins MxiG and MxiJ (Morita‐Ishihara et al., 2006). It has been proposed that the sorting platform consists of a central hub (Spa47) and six spokes (MxiN), with a pod‐like structure (Spa33) at the terminus of each spoke (Hu et al., 2015). Inactivation of the s*pa33* gene results in the absence of the cytoplasmic complex, no needles at the surface and consequently lacks protein secretion (Hu et al., 2015; Morita‐Ishihara et al., 2006). Spa33 exhibits sequence similarities with orthologs in other T3SSs including the flagellar proteins FliM and FliN of *Salmonella*, SpaO of *Salmonella,* and YscQ of *Yersinia*. As shown for SpaO and YscQ, an internal translation start codon is present in *spa33*, and leads to the expression of a short carboxy‐terminal variant, called Spa33^C^ (12‐kDa), which interacts with the full‐length protein (Spa33^FL^, 33‐kDa) (Bzymek, Hamaoka, & Ghosh, 2012; McDowell et al., 2016; Song et al., 2017). Absence of Spa33^C^ completely abolishes T3S, showing that Spa33^C^ is crucial for secretion as already shown in other T3SS (Bzymek et al., 2012; McDowell et al., 2016; Song et al., 2017). However, the exact roles of Spa33^FL^ and Spa33^C^ in T3SS are still unclear.

In the present study, we strived to characterize the role of Spa33^FL^ and Spa33^C^ in the regulation of the T3SS secretion by creating a series of mutants. We presented evidence that multiple proteins result from the *spa33* gene, some being required for T3SS function. Moreover, we show that Spa33 plays a significant role in effector secretion and interacts directly with MxiC and MxiI.

## 2 MATERIAL AND METHODS

### 2.1 Bacterial strains and cultures

Bacterial strains and plasmids used in this study are listed in Appendix Table A1. Unless stated otherwise, we consistently used *Shigella flexneri* M90T (serotype 5a) strain as a parental strain during this study. *Shigella* strains were grown in Tryptic Soy Broth (TSB) at 37°C and phenotypically selected on Congo red (CR) (Meitert et al., 1991). *Escherichia coli* (*E. coli*) strains, Top10, and BL21 DE3, were grown in Luria‐Bertani (LB) broth. When required, appropriate antibiotics with following final concentrations were added to the bacterial cultures: zeocin 50 μg/ml, kanamycin 50 μg/ml, streptomycin 100 μg/ml, ampicillin 100 µg/ml, and chloramphenicol 25 μg/ml for *E. coli* strains and 3 μg/ml for *Shigella* strains.

### 2.2 Construction of the spa33 and mxiCspa33 mutants

Generation of ∆*spa33* and ∆*mxiC*∆*spa33* mutants was achieved by single‐step gene inactivation method using the λ Red system as described previously (Datsenko & Wanner, 2000). Briefly, the coding sequence for the kanamycin cassette (Kan^R^) was amplified by PCR from pUC18K. The DNA fragments containing 600 bp of upstream and downstream regions of *spa33* gene were amplified by PCR using *S. flexneri* M90T virulence plasmid DNA as a template. The amplified DNA of *spa33* from the upstream and downstream regions was then assembled with the amplified Kan^R^ cassette by standard assembly PCR, according to manufacturer guidelines (New England Biolabs). The resulting amplicon was then transformed into *S. flexneri* M90T strain expressing λ Red recombinase from the pKD46 plasmid to replace *spa33*. The Kan^R^ transformants in which the recombination had occurred were selected on TSB agar plates containing kanamycin. Subsequently, *∆spa33* clones were further confirmed by PCR and sequence analysis of the amplified regions flanking the cassette. A similar approach was used to construct the *mxiCspa33* double mutant in the *∆mxiC* background (Botteaux et al., 2009).

### 2.3 Generation of recombinant plasmids and mutagenesis

All the plasmids and primers used in this study are listed in Appendix Tables A1 and A2, respectively. The plasmid pMK1 (pSU18‐*spa33*) was used to complement the *spa33* mutant. The gene encoding Spa33 was amplified using primers tailed with *BamH*I*/Hind*III restriction sites. The double digested PCR product with *BamH*I*/Hind*III restriction enzymes was ligated into the *BamH*I*/Hind*III sites of the low‐copy vector pSU18 (Invitrogen). To complement the *mxiC spa33* double mutant, we constructed a plasmid, carrying both native MxiC and Spa33 (pMK2) by cloning the *spa33* gene with 5′ insertion of the Shine and Dalgarno (*SD*) sequence into *KpnI/PstI* restriction sites of the pSU18‐MxiC (Cherradi et al., 2013)*.* In both constructs, a hexa‐histidines motif was fused to COOH‐terminal part of the Spa33 that facilitated detection of protein expression. To create specific site‐directed amino acid mutations, we applied the standard mutagenic PCR technique according to the TaKaRa PrimeSTARⓇ HS Premix Mutagenesis kit (TaKaRa Bio Inc) using the relevant specific primers listed in Appendix Table A2. Mutations were confirmed by sequencing (Eurofins).

To combine the expression of Spa33 variants, lacking either Spa33^C^ or Spa33^CC^, and Spa33^C^ *in trans*, we constructed pBAD‐Sap33^C^. The DNA sequence coding for the Sap33^C^ was amplified by PCR and digested with *Nco*I*/Hind*III*.* The resulting cleaved product was then inserted into the pBAD vector giving rise to pMK11. A series of single amino acid substitutions on pSU18‐Spa33 and pSU18‐MxiC‐Spa33 was generated and co‐expressed *in trans* with Spa33^C^ from pBADHisA.

All the plasmids expressing GST fusion and His fusion proteins used for the protein‐protein interaction assays are listed in Appendix Table A1. Plasmids expressing GST fused to MxiC, MxiC^F206S^, Spa33, Spa33^C^, and MxiI were constructed by inserting respective PCR excised DNA fragments of the sequences coding for *mxiC*, *mxiC^F206S^, spa33*, *spa33^C^*, and *mxiI*, respectively, into pGEX4T1. Plasmids expressing His recombinant proteins fused with His‐tag at its NH2‐terminal end (His‐Spa33) or both ends (His‐Spa33‐His) were constructed by inserting PCR digested DNA fragments encompassing the *spa33* coding sequence into the vector pET30a (+). Plasmid pMK25 (Spa33‐His) was constructed by cloning a PCR fragment carrying sequence encoding *spa33* into the vector pBADHisA. Site‐directed mutagenesis was used to create all subsequent point mutations according to the TaKaRa PrimeSTARⓇ HS Premix Mutagenesis kit (TaKaRa BioInc). All recombinant plasmids were sequenced (Eurofins).

### 2.4 Secretion tests

The detailed procedure for the preparation of crude extracts, leakage of the Ipa proteins into the culture supernatant and CR‐induced protein secretion were described in previous works (Allaoui, Sansonetti, & Parsot, 1992; Botteaux et al., 2009). *S. flexneri* strains were grown overnight at 37°C in TSB medium with appropriate antibiotics. The overnight cultures were diluted to an optical density at 600 (OD_600_) of 0.1 in 15 ml of TSB supplemented with appropriate antibiotics and grown at 37°C. In the case of *S. flexneri* strains carrying pBAD‐Spa33 or its derivatives, 0.001% arabinose was added to the culture when they reached an OD_600_ of 0.6. Cultures were grown to OD600 of ≅2, and bacteria were collected by centrifugation at 2,800 *g* for 15 min at 37°C. The supernatants were collected and precipitated with 4.5 g of ammonium sulfate for overnight as described previously (Botteaux et al., 2009). The bacterial pellet was suspended in 1X phosphate buffer saline (PBS) containing 200 μg/ml CR and induced for 20 min at 37°C on a shaker incubator. After incubation, bacteria were centrifuged at 13,000 *g* for 15 min at RT and supernatants were collected. The CR induced and noninduced samples were mixed with 4× Laemmli sample buffer, resolved on 12% SDS‐PAGE, and visualized by Coomassie blue staining or Western blot. All secretion tests were conducted at least three times.

### 2.5 GST‐pulldown assays

The *E. coli* BL21^DE3^ or Top10 strains were used in this study as the host cell for the expression of recombinant (GST and His fused) proteins. All the plasmids expressing GST, GST‐MxiC, GST‐MxiC^F206S^, GST‐Spa33^C^, GST‐MxiI, His‐tagged Spa33, and its derivatives used for the protein–protein interaction assays are listed in Appendix Table A1. To express the recombinant proteins, the cells were propagated in the LB medium containing appropriate antibiotic at 37°C and 200 rpm. Once the bacterial growth reached 0.6–0.7 at OD_600_, the protein expression was induced by adding isopropyl β‐D‐1‐thiogalactopyranoside (IPTG) to a final concentration of 0.1 mmol/L and incubating at 30°C for at least 3 hr. In the case of strains carrying pBAD‐Spa33^C^, 0.002% L‐arabinose was used for the induction. After 3 hr, cells were harvested by centrifugation (8,000 *g*, 15 min, 4°C). The harvested cells were resuspended in cold phosphate‐buffered saline (PBS) supplemented with 0.1% TritonX‐100, 0.15 mM PMSF, and iodoacetamide. Sonication was used to lyse the cell suspensions at the following settings: amplitude, 70; time, 3 min; pulsar, 10 s. Cell lysates were then clarified by centrifugation at 8,000 *g* for 30 min at 4°C. The cleared lysates were mixed with 200 µl of GST‐Bind™ Resin (EMD Millipore Novagen) which had been previously equilibrated with PBS buffer and incubated for one hour at room temperature (RT) while shaking. GST beads were recovered by centrifugation and then washed five times with PBS. Then lysates of target proteins with His‐tag expressed from *E. coli* strains (Rosetta DE3) were applied to the beads, which was followed by overnight incubation at 4°C. Beads were washed again extensively, and the captured proteins were eluted by incubating beads for 20 min at RT with elution buffer (40 mmol/L Tris pH 8.0, 500 mmol/L NaCl, and 50 mmol/L reduced glutathione). Eluted samples were separated by SDS‐PAGE and analyzed by Coomassie blue staining and Western blot.

### 2.6 Western blots

The following primary antibodies were used for hybridization: anti‐Histidine monoclonal antibody (Sigma‐Aldrich), anti‐Glutathione‐S‐Transferase (Sigma‐Aldrich), using anti‐IpaB (Barzu et al., 1993), anti‐IpaC (Phalipon et al., 1992), anti‐IpaD (Ménard, Sansonetti, & Parsot, 1993), anti‐IcsB (Kayath et al., 2010), anti‐IpaA (Tran Van Nhieu, Ben‐Ze'ev, & Sansonetti, 1997), anti‐MxiC (Botteaux et al., 2009). Secondary antibodies used were peroxidase‐conjugated anti‐rabbit IgG produced in goat, anti‐mouse IgG antibody produced in goat (Sigma‐Aldrich). Western blots were visualized on an Amersham Imager 600 (GE healthcare).

### 2.7 MS analysis

The bands corresponding to alternative Spa33 variants were excised from the stained gel and subjected to tryptic digestion. Briefly, the gel bands were incubated in 30 µl of 25 mM NH4HCO3, then reduced with 10 mM DL‐dithiothreitol during 30 min at 56°C and alkylated with 55 mM iodoacetamide during 20 min at room temperature. After, proteins were digested overnight with 2 µg of trypsin (Promega®, Belgium) at 37°C. After gel shrinking with acetonitrile, formic acid was added to 1% (v/v), and peptides were purified using StageTips C18 (Thermo Fischer Scientific®) according to the manufacturer's instructions. The samples were evaporated to dryness in a vacuum centrifuge and resuspended in 15 µl of 5% ACN/0.1% HCOOH. 5 µl‐aliquot of digested proteins was injected for mass spectrometric analysis.

Mass spectra were acquired using an AB Sciex 5600 Triple TOF mass spectrometer (AB Sciex®) interfaced to an Eksigent NanoLC Ultra 2D HPLC System (Eksignet®). Peptides were injected and concentrated on a trapping column (Waters Symmetry® C18 NanoAcquity 2G v/v, 20 mm × 180 µm, 5 µm) with a loading solvent (5% CAN/ 0.1% HCOOH). After 10 min, peptides were separated on a separation column (Waters Acquity® UPLC HSS T3, 250 mm × 75 µm, 1.8 µm) using a two steps acetonitrile gradient (5%–25% ACN/ 0.1% HCOOH in 60 min then 25%–60% CAN/ 0.1% HCOOH in 40 min) and were sprayed online in the mass spectrometer. MS1 spectra were collected in the range 400–1,200 *m*/*z* for 250 ms. The 20 most intense precursors with charge state 2–4 were selected for fragmentation, and MS2 spectra were collected in the range 100–2,000 *m*/*z* for 100 ms; precursor ions were excluded for reselection for 12 s.

## 3 RESULTS

### 3.1 The spa33 gene encodes 5 fragments

**Figure 1:**
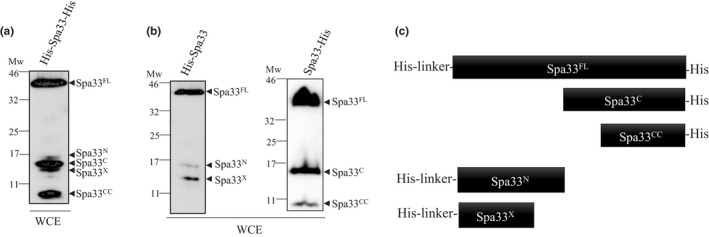
Analysis of the expression profile of different Spa33 fusion proteins. (a) Whole cell extracts (WCE) of *E. coli* (BL21) expressing His‐Spa33‐His were analyzed by Western blot using anti‐His monoclonal antibodies. (b) Whole cell extracts (WCE) of *E. coli* expressing His‐Spa33 (BL21) or Spa33‐His (TOP10) were analyzed by Western blot using anti‐His monoclonal antibodies. (c) Schematic representation of all the different fragments detected by WB depending of the His‐tag position. Mw: molecular weight in kDa

**Figure A1:**
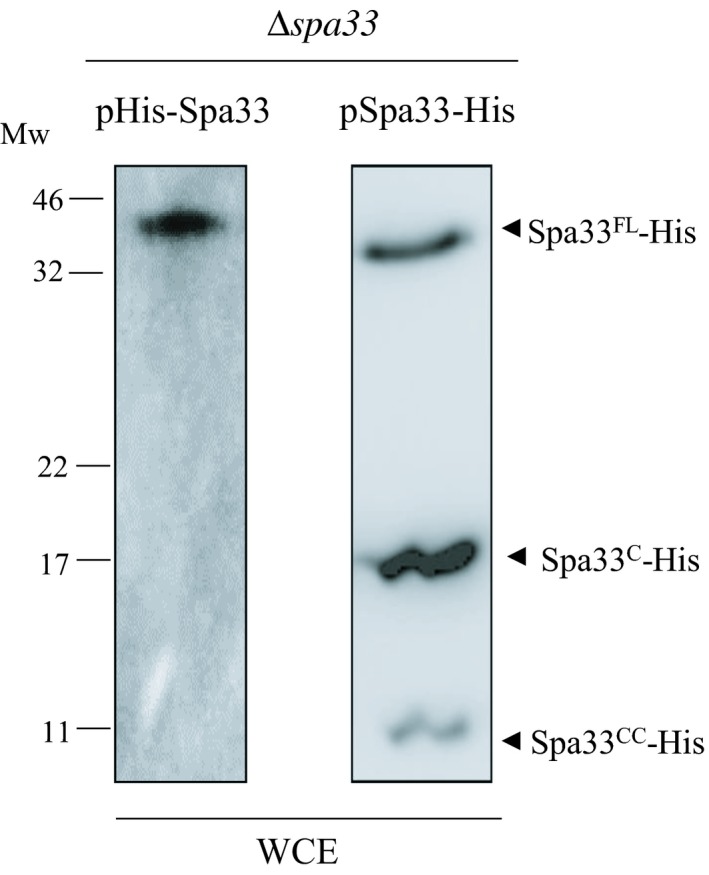
Expression profile of His‐Spa33 and Spa33‐His in *Shigella (∆spa33)*. Whole cell extract (WCE) of *∆spa33* producing His‐Spa33 (pHis‐Spa33) or Spa33‐His (pSpa33‐His) from a pBAD promoter were analyzed by western blot using anti‐His monoclonal antibody. Mw, molecular weight in kDa

To better understand the role of Spa33 in T3S, we cloned the *spa33* gene in an expression vector (pET30a), allowing fusion of a hexa‐histidine peptide to both ends of the protein (His‐Spa33‐His). By analyzing whole cell extracts with anti‐his antibodies, we observed 5 fragments of different sizes (Figure 1a). Three of these peptides were well expressed: ~33 kDa corresponding to the Spa33 full‐length (Spa33^FL^), ~12 kDa, which was previously discovered by McDowell et al. (2016), named Spa33^C^, and a smaller peptide of ~7 kDa (called hereafter Spa33^CC^) (Figure 1a and Table 1). The two other peptide fragments were barely detectable; one of ~11 kDa (called Spa33^N^) and another of ~10 kDa (called Spa33^X^) (Figure 1a). To further characterize the nature of these different peptides, we constructed plasmids encoding recombinant Spa33 proteins fused to a His‐tag at their NH2‐terminal (His‐Spa33) or to their COOH‐terminal end (Spa33‐His) (Figure 1b,c). Analysis of His‐Spa33 expression profile showed the presence of Spa33^FL^, Spa33^N^, and Spa33^X^ (Figure 1b and Table 1) while Western blot analysis of Spa33‐His expression only detected Spa33^FL^, Spa33^C^, and Spa33^CC^ (Figure 1b). Since the 5 fragments are only detectable if expressed from a strong promoter (pET30a), they could not be visualized in the *Shigella* background (*spa33* mutant). Indeed, expression of His‐Spa33 or Spa33‐His from a pBAD plasmid showed the presence of Spa33^FL^, Spa33^C^, and Spa33^CC^, but no Spa33^N^ or Spa33^X^ (Appendix Figure A1).

**Table 1:** Summary of different fragments produced by *spa33* and their role in T3SS

Name of the fragment	Mechanism of production	Molecular weight (in kDa)	Role in T3SS
Spa33^FL^	Full length protein	33.4	Required
Spa33^C^	Internal start codon (V192)	11.6	Required
Spa33^CC^	Internal start codon (M237)	6.5	Required
Spa33^N^	Transcriptional slippage site in spa33 (180−189p)	11.5	Not required (in vitro)
Spa33^x^	Unknown	~10	Unknown

### 3.2 Spa33^CC^ is a product of an alternative start codon in the spa33 gene and is necessary for T3SS formation

**Figure 2:**
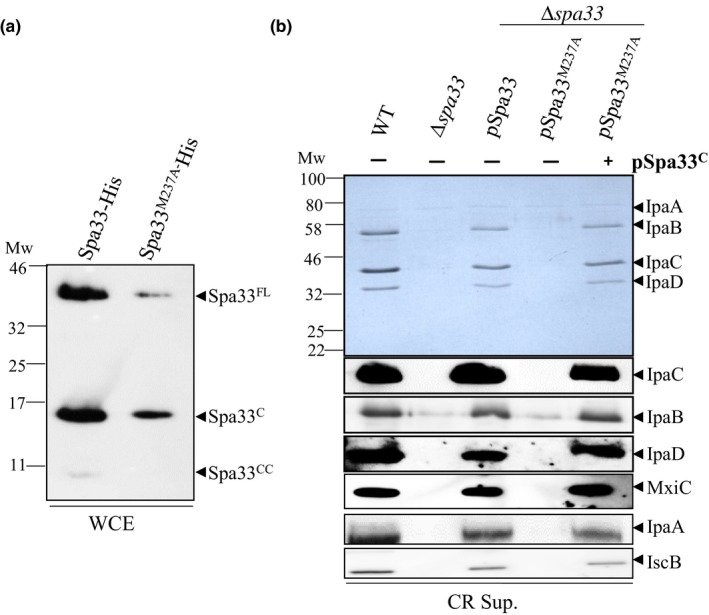
Spa33^CC^ arises from an alternative start codon and is required for protein secretion. (a) Whole cell extracts (WCE) of the *E. coli* strains (TOP10) harboring plasmid expressing Spa33‐His or its mutated derivative (Spa33^M237A^) were analyzed by immunoblotting with anti‐His antibodies. (b) Coomassie blue staining (upper panel) or Western blot (lower panels) analysis of secreted proteins by the wild‐type strain (WT), the spa33 mutant (*∆spa33),* the *spa33* mutant complemented with pSU18‐spa33 (pSpa33) or its mutated derivative (pSpa33^M237A^), with or without Spa33^C‐CC^ (+ or − pSpa33^C^), under CR induction (CR sup.) and using IpaC, IpaB, IpaD, MxiC, IpaA, and IscB antibodies. Mw: molecular weight in kDa

**Figure A2:**
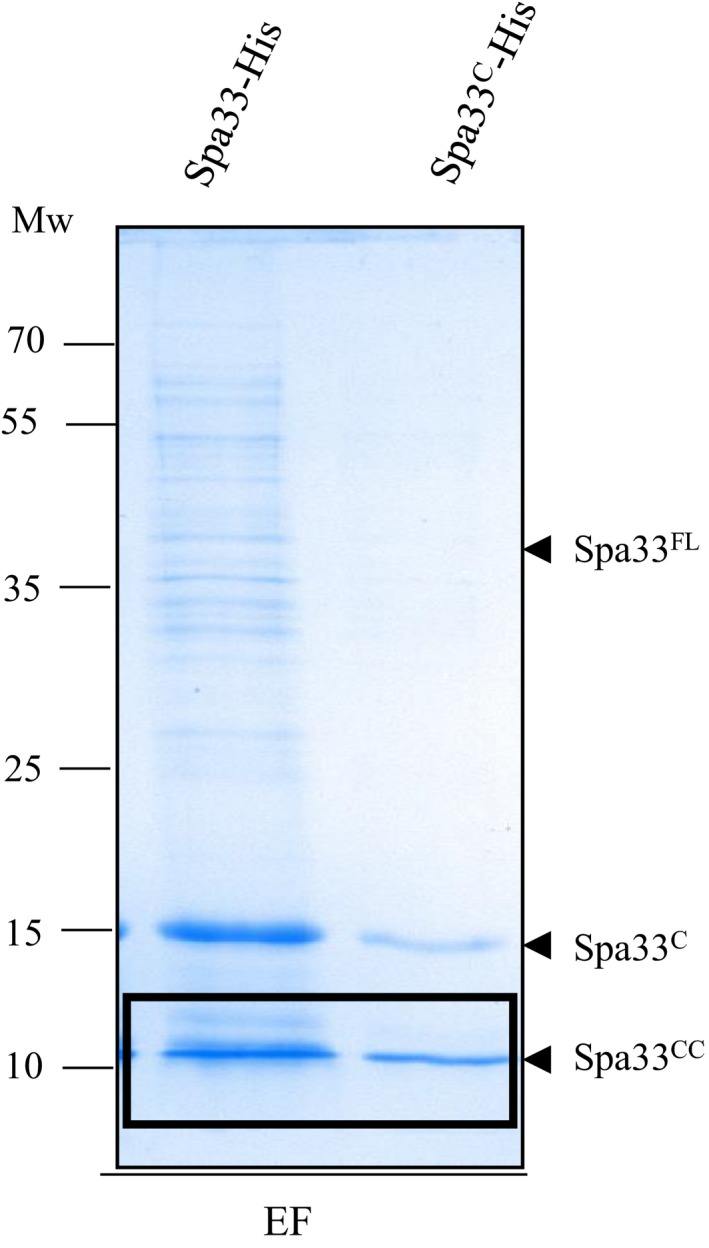
Purification of Spa33‐His and Spa33^C^‐His in *E. coli*. Spa33‐His and Spa33^C^‐His were over expressed in *E. coli* (Top10) and purified using His Cobalt Resin. Eluted fractions (EF) were separated on 15% SDS‐PAGE and visualized by Coomassie blue staining. Spa33^CC^ was excised from the gel and analyzed by MS. Mw: molecular weight in kDa

As Spa33^CC^ is only detected by COOH‐terminal His‐tag, we first assessed by mass spectrometry (MS) if this fragment was part of the COOH‐terminal domain of Spa33. After purification by His‐trap, SDS‐PAGE, and trypsin digestion, we observed that the sequenced peptides cover the residues 244 to 292 (Appendix Table A3 and Figure A2). Based on the apparent size of Spa33^CC^ (7‐kDa) and our MS data, we searched for potential alternative start codons in *spa33*. We mutated an ATG codon, encoding the M237 residue in Spa33, by replacing it with an alanine (Spa33^M237A^). Study of the protein expression showed that M237A mutation totally abolished expression of Spa33^CC^ (Figure 2a) suggesting that M237 acts as an internal translation start codon for Spa33^CC^. Insertion of a stop codon upstream of the start codon of the Spa33^CC^ (M237) fragment within the *spa33* gene still allows Spa33^CC^ production, confirming the presence of an alternative start codon for Spa33^CC^ (Appendix Figure A4).

**Figure A3:**
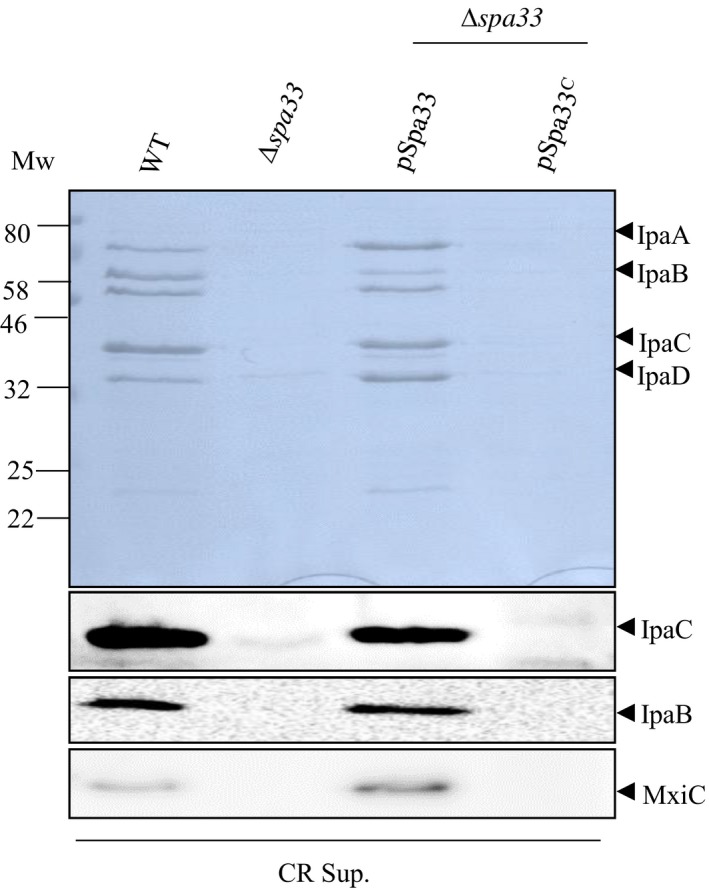
Spa33^C^ alone is not able to complement *Δspa33*. Supernatants of induced culture (CR Sup.) of the wild‐type strain (WT), the *spa33* mutant (∆*spa33*), the *spa33* mutant complemented by pBAD‐Spa33 (pSpa33) or pBAD‐Spa33^C^ (pSpa33^C^) were analyzed by Coomassie blue staining (upper panel) or Western blot (lower panels) using anti‐IpaC, anti‐IpaB, and anti‐MxiC antibodies. Mw, molecular weight in kDa

We generated the same mutation on a low copy plasmid (pSU18) carrying the *spa33* gene (pSpa33), which can restore proteins secretion in a *spa33* knockout mutant (Δ*spa33*) (Figure 2b). To analyze the secretion profile in the absence of Spa33^CC^, we induced secretion by adding Congo Red dye (CR), a small amphipathic molecule, which mimics host cell contact (Meitert et al., 1991). Western blot analysis of supernatant from CR‐induced cultures showed that absence of Spa33^CC^ (pSpa33^M237A^) results in total lack of protein secretion (Figure 2b), including absence of translocators (IpaC, IpaB, and IpaD), secretion regulator (MxiC) and early effectors (IpaA and IcsB). Detection of the wild type or Spa33‐His variants by Western blot using anti‐His antibodies were unsuccessful probably due to the low expression rate from the pSU18 vector. Cloning of *spa33* in a medium copy plasmid with a stronger promoter (pBAD) also allows perfect complementation of the *spa33* mutant (Appendix Figure A3). The Spa33^M237A^ mutant generated on this plasmid presents the same phenotype as pSU18‐*spa33* (data not shown).

**Figure A4:**
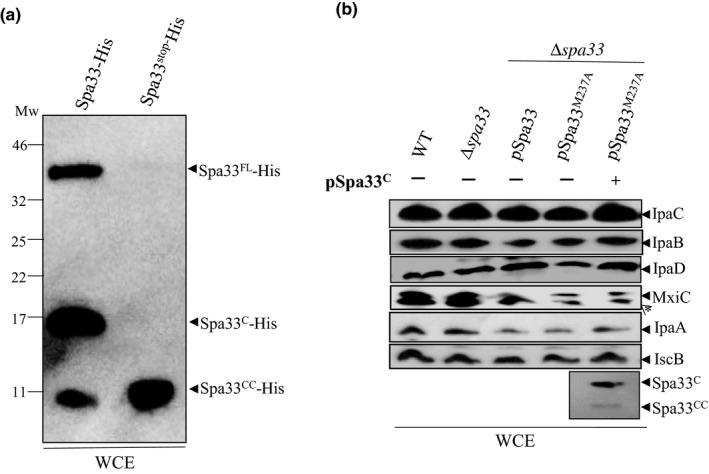
(a) Insertion of stop codon after the start codon for Spa33^C^ (V192) did not abolish the production of Spa33^CC^. Whole cell extracts (WCE) of the *E. coli* strains (Top10) harboring plasmid expressing Spa33‐His or its mutated derivative (Spa33^stop‐^His) were analyzed by Western blot using anti‐his monoclonal antibodies. (b) Absence of Spa33^CC^ did not impair virulence proteins production. Analysis of whole cell extracts (WCE) of the wild‐type strain (WT), the *spa33* mutant (∆*spa33*), the *spa33* mutant complemented by pSU18‐Spa33 (pSpa33) or its mutated derivative (pSpa33^M237A^), with or without Spa33^C‐CC^, resolved by SDS‐PAGE and analyzed by Western blot using anti‐IpaC, IpaB, IpaD, MxiC, IpaA, IscB, and His antibodies. Mw, molecular weight in kDa. *Nonspecific band

To understand if the absence of secretion was only due to the absence of Spa33^CC^, we restored expression of Spa33^CC^ by transforming a second plasmid (pBAD‐Spa33^C^‐His) that encodes Spa33^C^ and Spa33^CC^ in the *spa33* mutant expressing Spa33^M237A^ (*trans*‐complementation). This plasmid allows the expression of Spa33^C^ but also of Spa33^CC^ (Appendix Figure A4). We observed that trans‐complementation in the Δ*spa33*/pSpa33^M237A^ background restored the secretion to the WT level (Figure 2b).

### 3.3 Spa33^N^ is a slippage product from the spa33 gene but is not required for T3SS secretion

**Figure 3:**
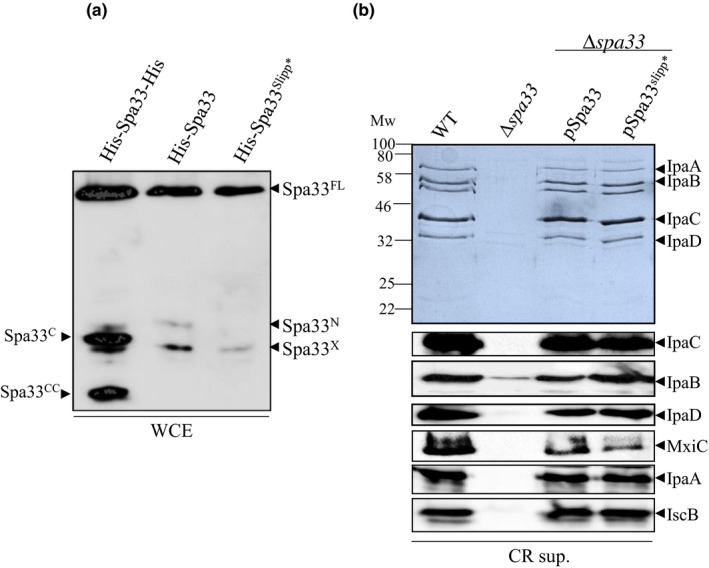
(a) Analysis of *E. coli* (BL21) expressing His‐Spa33‐His, His‐Spa33, and His‐Spa33^slipp*^ on SDS‐PAGE by western blot using anti‐His antibody. (b) Coomassie blue staining (upper panel) or Western blot (lower panels) analysis of secreted proteins by the wild‐type strain (WT), the *spa33* mutant (*∆spa33),* the *spa33* mutant complemented with pSU18‐spa33 (pSpa33) or its mutated derivative (pSpa33^slipp*^), under CR induction (CR sup.) and using IpaC, IpaB, IpaD, MxiC, IpaA, and IscB antibodies. Mw: molecular weight in kDa

**Figure A5:**
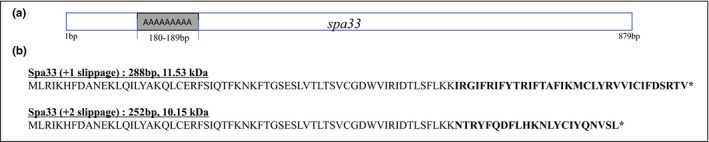
(a) Schematic representation of the s*pa33* gene and its slippage site (gray). (b) Hypothetical sizes and amino acid sequences of the two potential slippage products by +1 or +2 frame shifting. The sequence in amino acid which differs from Spa33 is written in bold

Work published by Penno *et al*. demonstrates that at the RNA level, a string of 9 alanines at position 180–189 bp of *spa33* allows low‐level transcriptional slippages (Penno et al., 2006). According to our in silico analysis, the molecular weight of Spa33^N^ (around 11 kDa), potentially corresponds to a protein produced during a +1 slippage (Appendix Figure A5). To check this hypothesis, we mutated the slippage site to prevent frameshifting and analyzed the expression profile of the resulting His‐Spa33^Slipp*^. This plasmid allows a better detection of both Spa33^N^ and Spa33^X^ than His‐Spa33‐His. Our results showed that the Spa33^N^ is totally absent in this mutant (Figure 3a) while Spa33^X^ is still produced although at a lower level. However, the mutation of the slippage site was not associated with any detectable change in T3SS secretion under induced condition (Figure 3b).

### 3.4 Role of Spa33^C^ in T3SS

**Figure 4:**
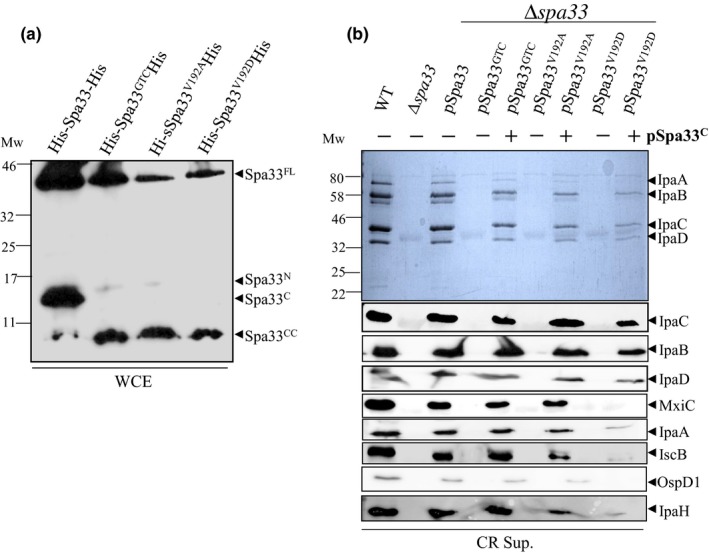
Simultaneous expression of Spa33^C^ and Spa33^V192D^ blocks effectors secretion. (a) Whole cell extracts (WCE) of *E. coli* (BL21) expressing Spa33 variants were analyzed by Western blot using anti‐His antibody. (b) Coomassie blue staining (upper panel) or Western blot (lower panels) analysis of secreted proteins by the wild‐type strain (WT), the *spa33* mutant (*∆spa33),* the *spa33* mutant complemented with pSU18‐spa33 (pSpa33) or its mutated derivatives (pSpa33^GTC^, pSpa33^V192A^, pSpa33^V192D^), with or without Spa33^C‐CC^ (+ or − pSpa33^C^), under CR induction (CR sup.) and using IpaC, IpaB, IpaD, MxiC, IpaA, IscB, OspD1, and IpaH antibodies. Mw: molecular weight in kDa

Spa33^C^ arises from an alternative translation start codon (GTG) which encodes a valine at position 192 in the full‐length protein (McDowell et al., 2016). To further investigate the role of Spa33^C^ in T3SS, we constructed 3 mutants by changing the alternative start codon by a synonymous mutation (Spa33^GTC^) or two nonsynonymous mutations, where V192 was replaced with an alanine (Spa33^V192A^) or with an aspartic acid (Spa33^V192D^). These mutations were first introduced in the His‐Spa33‐His plasmid to allow detection of all the Spa33 fragments. As expected, the three constructs allow expression of Spa33^FL^ but lack the Spa33^C^ product (Figure 4a). In the absence of Spa33^C^, we still detect Spa33^N^, though scarcely, but do not detect Spa33^X^ anymore.

We then generated the same mutations in pSpa33 and introduced the resulting plasmids (pSpa33^GTC^, pSpa33^V192A^, and pSpa33^V192D^) into the *spa33* mutant. Analysis of CR‐induced culture supernatants showed that, as expected, all these variants (McDowell et al., 2016), were unable to restore protein secretion compared to the complemented strain (Figure 4b). Same mutations were generated in pBAD‐Spa33‐His and transformed in the *spa33* mutant. They all present the same phenotype as the corresponding mutations in pSU18‐*spa33* (data not shown).

**Figure A6:**
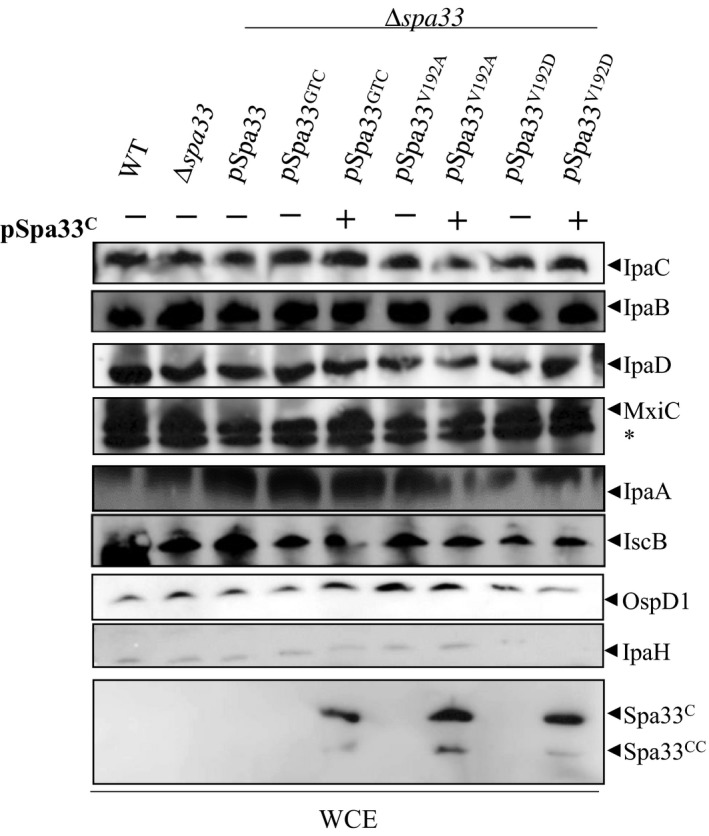
Whole cell extracts (WCE) of the wild‐type strain (WT), the *spa33* mutant (∆*spa33*), the *spa33* mutant complemented by pSU18‐Spa33 (pSpa33) or its mutated derivatives, with or without Spa33^C^ (pSpa33^C^), analyzed by Western blot using anti‐IpaC, IpaB IpaD, MxiC, IpaA, IscB, OspD1, IpaH, and anti‐His antibodies. *Nonspecific band

To understand if the absence of secretion was only due to the absence of Spa33^C^, we restored expression of Spa33^C^ by transforming a second plasmid expressing Spa33^C^ and Spa33^CC^ in the *spa33* mutant (*trans*‐complementation; Appendix Figure A6). Supernatants from CR induced cells (Figure 4) and whole cell extracts (Appendix Figure A6) were analyzed by Western blot using antibodies targeting different classes of secreted proteins. We observed that expression of Spa33^C^ with pSpa33^GTC^ or pSpa33^V192A^ allows secretion of proteins at wild‐type levels (Figure 4b). Interestingly, *trans*‐complementation in the presence of pSpa33^V192D^ strongly decreases secretion of MxiC and early effectors (IpaA and IcsB), while translocators (IpaC, IpaB, and IpaD) were secreted at wild‐type levels (Figure 4b). OspD1, an early effector and anti‐activator of the MxiE‐regulated genes is not secreted by the effector mutant Spa33^V192D+C^. Consequently, no late effectors (IpaH) were detected after CR induction (Figure 4b). This “effector mutant” phenotype, as previously described (Cherradi et al., 2013; El Hajjami et al., 2018; Kenjale et al., 2005), suggests a role of Spa33 in the regulation of the secretion hierarchy. Expression of Spa33^C^ alone in the *spa33* mutant does not allow T3 secretion (Appendix Figure A3).

### 3.5 Inactivation of mxiC or expression of MxiC^F206S^ in the effector mutant restores effector secretion

**Figure 5:**
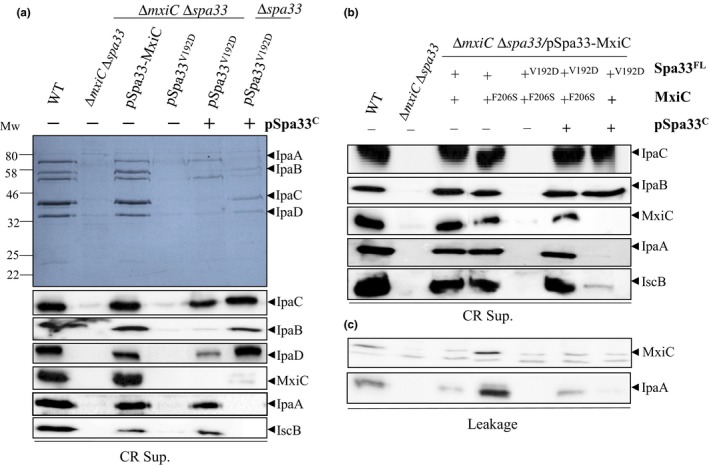
Inactivation of *mxiC* or co‐expression of MxiC^F206S^ in the Δ*spa33*/Spa33^C^+Spa33^V192D^ background restores effectors secretion. (a) Induced culture supernatants (CR Sup.) of the wild‐type strain (WT), the *spa33 mxiC* mutant (∆*spa33* ∆*mxiC*), the *spa33 mxiC* mutant complemented by pSU18‐Spa33‐MxiC (pSpa33‐MxiC) or its mutated derivatives, with or without Spa33^C^, were analyzed by Coomassie blue staining (upper panel) or Western blot (lower panels) using anti‐IpaC, anti‐IpaB, anti‐IpaD, anti‐MxiC, anti‐IpaA, and anti‐IscB antibodies. (b) Induced and (c) non‐induced culture supernatants (leakage) of the wild‐type strain (WT), the *spa33 mxiC* mutant (∆*spa33* ∆*mxiC*), the *spa33 mxiC* mutant complemented by pSU18‐Spa33‐MxiC (pSpa33‐MxiC) or its mutated derivatives, with or without Spa33^C‐CC^ (+ or − pSpa33^C^) were analyzed by Western blot using anti‐IpaC, anti‐IpaB, anti‐IpaD, anti‐MxiC, anti‐IpaA, and anti‐IscB antibodies. Mw: molecular weight in kDa

MxiC serves as an intracellular sorter retaining effectors before activation of secretion (El Hajjami et al., 2018). We reasoned that MxiC could block effector secretion observed in the *spa33* mutant expressing pSpa33^V192D^ and Spa33^C^. Thus, we inactivated the *mxiC* gene in this background and performed secretion assays. Western blot analysis of secreted proteins revealed that secretion of effectors (IpaA and IcsB) in the absence of MxiC was restored to wild‐type level (Figure 5a). Secretion of translocators is reduced in this background due to *mxiC* inactivation (Botteaux et al., 2009). Our results suggest that the V192D mutation in Spa33 impaired, directly or indirectly, the secretion of MxiC and its role in effector secretion regulation.

**Figure A7:**
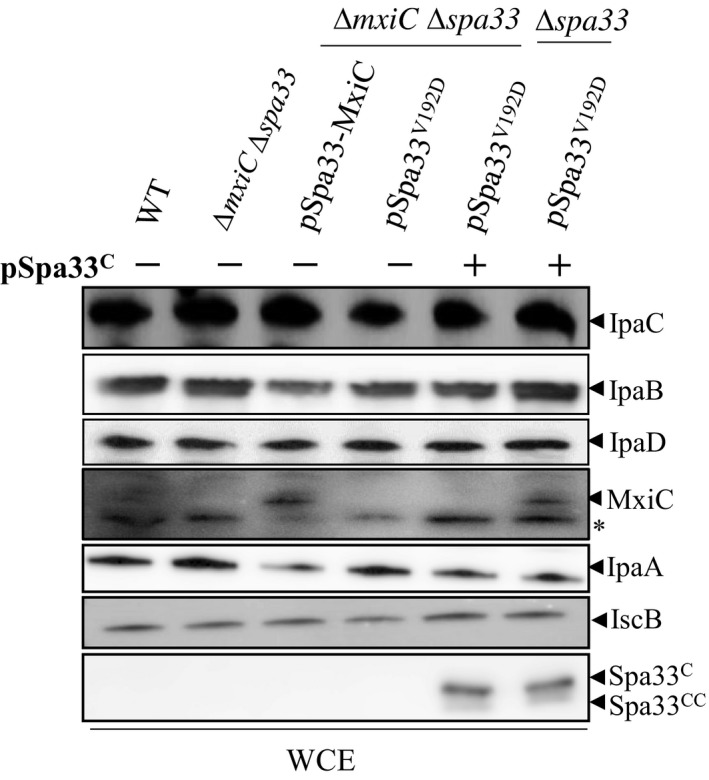
Whole cell extracts (WCE) of the wild‐type strain (WT), the *mxiCspa33* mutant (∆*mxiC*∆*spa33*), the *spa33mxiC* mutant complemented by pSU18‐Spa33‐MxiC (pSpa33‐MxiC) or pSU18‐Spa33^V192D^, with or without Spa33^C^ (pSpa33^C^), analyzed by Western blot using anti‐IpaC, IpaB, IpaD, MxiC, IpaA, IscB, and anti‐His antibodies. Expression of pSpa33^V192D^ and Spa33^C^ was used as a control. *Nonspecific band

**Figure A8:**
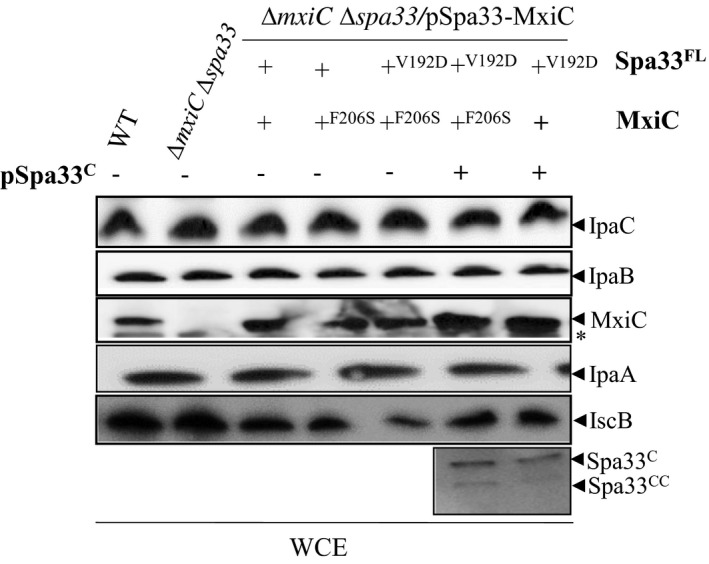
Whole cell extracts (WCE) of the wild‐type strain (WT), the *mxiCspa33* double mutant (∆*mxiC*∆*spa33*), the *spa33* mutant complemented by pSU18‐Spa33‐MxiC (pSpa33‐MxiC), or its mutated derivatives, with or without Spa33^C^ (pSpa33^C^), analyzed by Western blot using anti‐IpaC, IpaB, MxiC, IpaA, IscB, and anti‐His antibodies. *Nonspecific band

We have previously reported that a variant of MxiC (MxiC^F206S^) leads to a constitutive secretion of effectors (i.e., before induction), as observed in the *mxiC* mutant, probably due to an early secretion of MxiC^F206S^ (Cherradi et al., 2013). Expression of this variant in an “effector mutant” background restores effector secretion (Cherradi et al., 2013; El Hajjami et al., 2018). To investigate the capacity of the MxiC^F206S^ variant to rescue the “effector mutant” phenotype of Spa33^V192D^/Spa33^C^ *,* we constructed the pMxiC^F206S^‐Spa33 and pMxiC^F206S^‐Spa33^V192D^ vectors and co‐expressed them with Spa33^C^ in the double *mxiC spa33* mutant. We analyzed the ability of the *mxiC spa33* mutant and its derivatives to secrete virulence proteins under both constitutive (leakage) and induced conditions (CR). We observed that, when MxiC^F206S^, Spa33^V192D^, and Spa33^C^ were simultaneously expressed, effector secretion was restored, as observed in the absence of MxiC (Figure 5b). Interestingly, analysis of noninduced culture supernatants established that this mutant did not show an increased leakage as usually seen with MxiC^F206S^ (Figure 5c). Our results suggest that these two mutations, one on MxiC and one on Spa33, rescue each other's phenotypes and support that Spa33 is involved in T3SS regulation. Whole cell extracts analysis showed that the lack of secretion in some mutants is not due to impaired proteins production (Appendix Figures A7 and A8).

### 3.6 Spa33 is interacting with MxiC and MxiI

**Figure 6:**
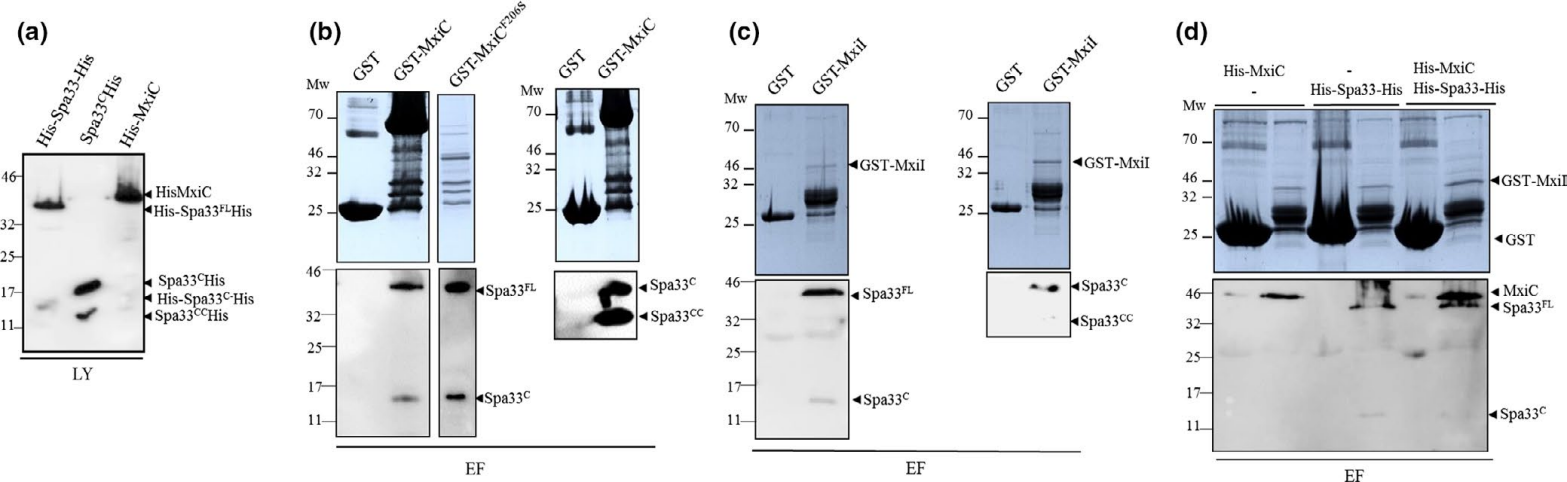
Spa33^C^ interact with MxiC and MxiI and forms a complex in vitro*.* (a) Analysis of the clarified lysates (LY) of His‐Spa33‐His or Spa33^C^‐His and His‐MxiC by immuno‐detection using anti‐his antibodies. Analysis of the eluted fractions (EF) by Coomasie blue staining (upper panels) and immuno‐detection using anti‐his antibodies (lower panels) of (b) GST‐MxiC or GST alone against His‐Spa33‐His or Spa33^C^‐His. (c) GST‐MxiI or GST alone against His‐Spa33‐His or Spa33^C^‐His and (d) GST‐MxiI or GST alone against both His‐Spa33‐His and His‐MxiC. Mw: molecular weight in kDa

As our previous results suggest a (direct or indirect) link between Spa33 and MxiC, we tested the potential interaction between these two proteins. GST‐MxiC was immobilized on glutathione‐sepharose beads and incubated with *E. coli* lysates expressing His‐Spa33‐His (Figure 6a). In lysates, only two forms of Spa33 were detectable: Spa33^FL^ and Spa33^C^, suggesting that the other fragments are not soluble. Subsequently, fractions eluted were analyzed by Western blot, using anti‐His antibody. As shown in Figure 6b, Spa33^FL^ and Spa33^C^ copurified with GST‐MxiC. We further tested the interaction between GST‐MxiC^F206S^ and Spa33 which was unaffected, even if GST‐MxiC^F206S^ is less stable/soluble (Figure 6b).

To test the ability of Spa33^CC^ to bind to MxiC, we produced it from another plasmid encoding Spa33^C^ and Spa33^CC^ only. Indeed, the latter plasmid allows the production of a higher quantity and more soluble form of Spa33^CC^ (Figure 6a). Our results showed that Spa33^C^ and Spa33^CC^ co‐eluted with GST‐MxiC, in the absence of Spa33^FL^ (Figure 6b).

The interaction between Spa33 and the gatekeeper protein MxiC prompted us to investigate whether Spa33 interacts with other proteins, implicated in the regulation of effectors secretion. In *Shigella*, the inner‐rod component, MxiI, interacts with MxiC to prevent effectors secretion (Cherradi et al., 2013; El Hajjami et al., 2018). Cleared lysates prepared from *E. coli* producing His‐Spa33‐His and Spa33^C^‐His were incubated with GST‐MxiI or GST alone, which had been preincubated with glutathione‐sepharose beads. We found that Spa33^C^‐His, produced either with Spa33^FL^ or with Spa33^CC^, interacts with GST‐MxiI (Figure 6c).

As Spa33 can interact with both MxiC and MxiI, we hypothesized that they could form a complex. Glutathione‐sepharose beads coated with either GST alone or GST‐MxiI were incubated with a premix of His‐MxiC and His‐Spa33‐His lysates (Figure 6d). Both His‐Spa33‐His and His‐MxiC were detected in the elution fractions with anti‐His antibody confirming that MxiI, MxiC, and Spa33 can form a complex in vitro (Figure 6d).

### 3.7 Spa33^V192D^ mutation did not abolish interaction with Spa33^C^

As the expression of Spa33^V192D^ and Spa33^C^ allows translocator secretion but not that of effector, we wanted to test if the mutation had any effect with respect to binding of Spa33^C^, MxiC, or MxiI. We cloned the COOH‐terminal part of *spa33* in a plasmid allowing its NH2‐terminal fusion with a GST‐tag (pGEX4T1‐Spa33^C^) and tested for the interaction with His‐Spa33‐His, His‐Spa33^GTC^‐His, or His‐Spa33^V192D^‐His.

**Figure 7:**
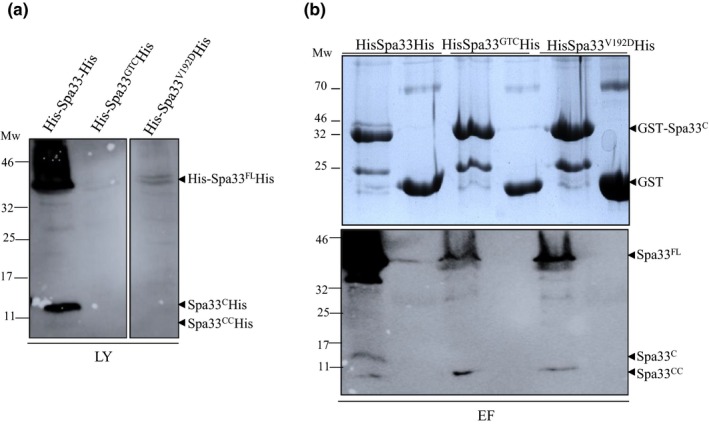
Spa33^V192D^ mutation did not abolish interaction with Spa33^C^. Cell suspensions of *E. coli *(BL21) producing His‐Spa33‐His, His‐Spa33^GTC‐^His, and His‐Spa33^V192D^‐His were mixed with cell suspensions of GST‐Spa33^C^ or GST alone followed by sonication and centrifugation. Clarified lysates were incubated with glutathione‐sepharose beads and proteins were eluted as described in experimental procedure. (a) Analysis of clarified lysates (LY) by Western blot using anti‐His antibodies. (b) Analysis of eluted fractions (EF) by Coomassie blue staining (upper panel) or immunoblot using anti‐His antibodies (lower panel). Mw: molecular weight in kDa

We know that Spa33^FL^ is less stable in the absence of Spa33^C^ (Figure 4a and McDowell et al., 2016). We failed to detect Spa33^FL^ in the lysate in the absence of Spa33^C^ suggesting that Spa33^C^ also has a role in solubilization of Spa33^FL^. To overcome this problem, we expressed all recombinant proteins separately and then mixed cell suspensions before sonication. The premixes of clarified cell lysates (Figure 7a) were then immobilized on glutathione‐sepharose beads and washed. Analysis of eluted fractions revealed that the Spa33^V192D^ mutation did not affect the ability of Spa33^FL^ to bind to Spa33^C^ (Figure 7b). We could observe Spa33^CC^ in all the eluted fractions but not in the lysates, as previously observed (Figure 6a).

**Figure A9:**
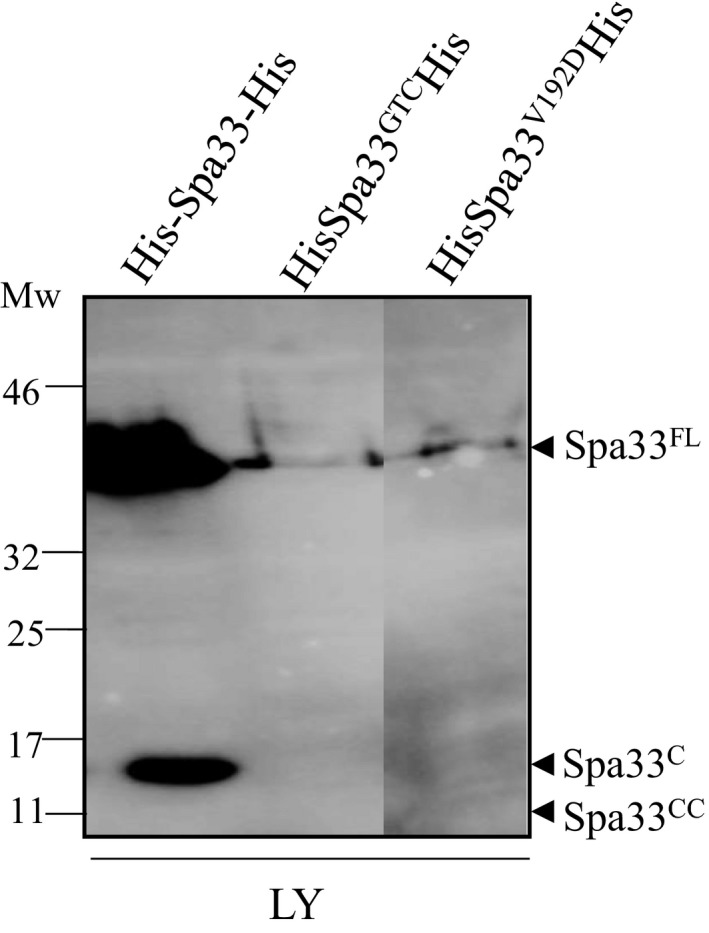
Analysis of lysates (LY) of *E. coli expressing* His‐Spa33‐His, His‐Spa33^GTC^ and His‐Spa33^V192D^ premixed with GST‐MxiC by Western blot using anti‐His antibodies. Mw: molecular weight in kDa

Since the previously observed deficiency in effectors secretion could be due to changes in the interaction of Spa33^V192D^ with MxiC and/or MxiI, we tested the potential interaction between GST‐MxiC, GST‐MxiI, and His‐Sap33^V192D^‐His. Unfortunately, the expression of Spa33^FL (GTC or V192D)^ in the absence of Spa33^C^ was barely detectable in the cell lysates and not sufficient to work with, even when premixed with GST‐MxiI or GST‐MxiC (Appendix Figure A9 and data not shown).

## 4 DISCUSSION

In this study, we showed that Spa33, a C‐ring component, plays a significant role in the regulation of secretion and encodes multiple proteins from a single gene, namely Spa33^FL^, Spa33^C^, Spa33^CC^, Spa33^N^, and Spa33^X^ (Table 1).

**Figure A10:**
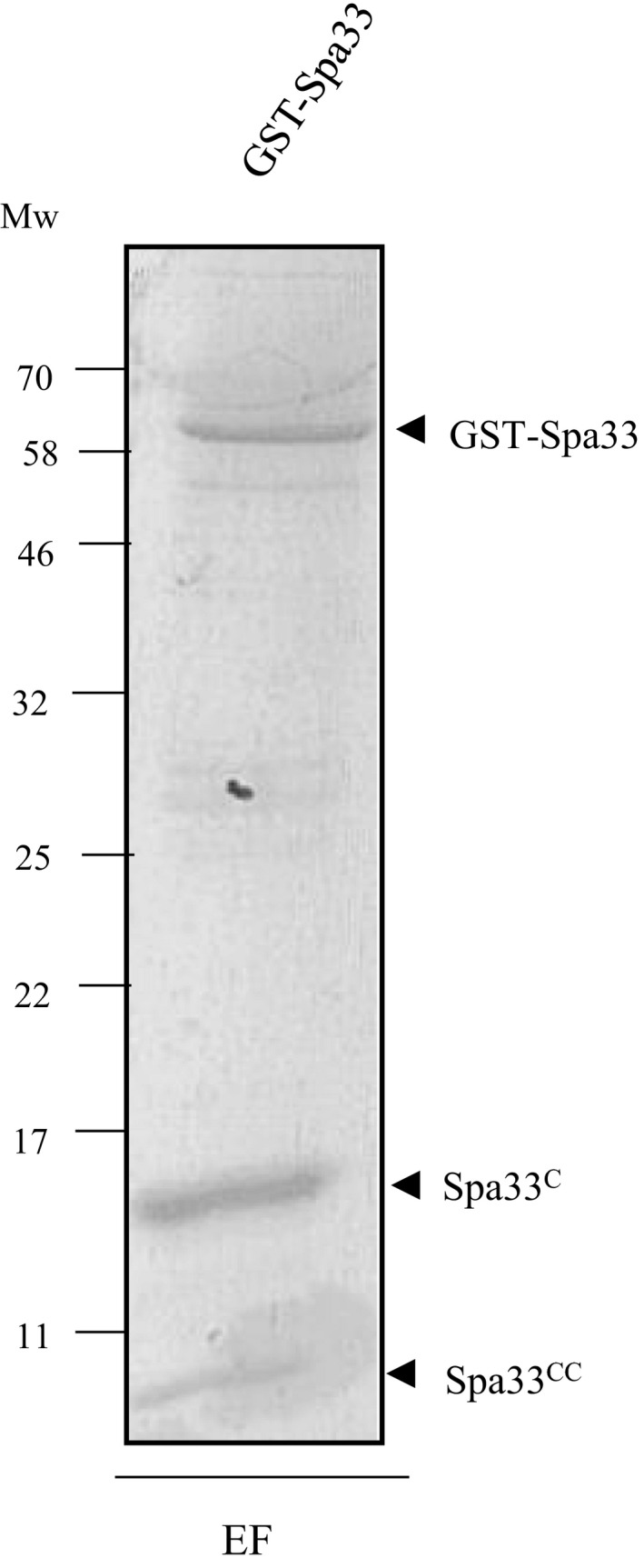
Purification of GST‐Spa33. GST‐Spa33 was produced in *E. coli* (Top10), and cell lysates were incubated with glutathione‐sepharose beads. Eluted fractions (EF) were separated on SDS‐PAGE and analyzed by Coomassie blue staining. Mw: molecular weight in kDa

For the first time with Spa33 homologous proteins, we have identified an internal start codon, encoding a methionine at position 237 and leading to a small ~7 kDa fragment. The absence of Spa33^CC^ results in the lack of T3SS substrate secretion indicating that this fragment is critical for T3SS function. It is noteworthy that lack of either Spa33^C^ or Spa33^CC^ mimics the knockout mutant phenotype in terms of protein secretion. Therefore, we cannot exclude the possibility that Spa33^CC^ and Spa33^C^ also contribute to T3SS assembly. Spa33^CC^ is not expressed significantly and we failed to find a ribosomal binding site (Shine‐Dalgarno sequence) upstream of the M237. Spa33^FL^ and Spa33^C^ were shown to interact with each other to form a complex in a 1:2 ratio, and further oligomerize in a defined order to form a functional cytoplasmic complex structure (McDowell et al., 2016). However, our results showed that Spa33^CC^ also copurified with Spa33^FL^ and Spa33^C^ (Appendix Figure A10), indicating that Spa33^CC^ is also part of Spa33^FL^‐Spa33^C^ complex. Spa33^X^, the only fragment we failed to find an origin for, seems linked, directly or indirectly, to Spa33^CC^ expression as the fragment totally disappeared in the absence of Spa33^CC^. A more detailed study of compositional and conformational changes of these protein assemblies under various conditions may provide a better insight to understand the complex regulatory dynamics involved.

Spa33^N^ was identified as a slippage product of the *spa33* gene. This fragment is barely expressed and therefore barely detected in whole cell extracts and not detected at all in soluble fractions. The rate of slippage events from *spa33*, measured by Penno *et al*., was <14% of total RNA, which can explain the difficulties encountered to detect and purify this fragment. We failed to find a role for Spa33^N^ in T3SS under our experimental conditions. However, it is well‐known that transcription of *spa33* is repressed by fumarate and nitrate reductase (FNR) binding in the absence of oxygen (O_2_), leading to T3SS functional impairment (Marteyn et al., 2010). Hence, Spa33^N^ could have a role in regulation under different conditions that bacteria could encounter during the infection process and not represented in our experimental design. Future work to identify the exact role of Spa33^N^ is needed as it appears to be a specific feature of *Shigella's* T3SS regulation, as the homologous genes (*yscQ*, *spaO*) do not harbor any slippage sites in their genes (data not shown).

Spa33^FL^ and Spa33^C^ have been previously shown to be important for T3SS (McDowell et al., 2016) as the absence of Spa33^C^ lead to a total deficiency in secretion. Nevertheless, the mechanism by which Spa33^C^ plays a role in secretion remains unclear. We have shown that expression of Spa33^C^ *in trans* could restore secretion in the Spa33^GTG^ variant. This result is not surprising as the homologous proteins of Spa33^FL^ and Spa33^C^ are encoded by separate genes in other systems (i.e., *fliM* and *fliN* genes in *Salmonella flagella*). More surprisingly, *trans*‐complementation with Spa33^C^ in the Spa33^V192D^ background, also lacking Spa33^C^ but harboring a point mutation on the full‐length form, restored secretion of translocators, but not of effectors. In several mutational studies on MxiH and MxiI, both implicated in signal transmission, this phenotype has already been described (Cherradi et al., 2013; Kenjale et al., 2005; Martinez‐Argudo & Blocker, 2010; Roehrich, Guillossou, Blocker, & Martinez‐Argudo, 2013). In the mutants MxiH^K69A^ and MxiI^Q67A^, the lack of effector secretion was shown to be dependent on the presence of MxiC as *mxiC* inactivation in these strains restores effector secretion to wild‐type levels (Cherradi et al., 2013; El Hajjami et al., 2018). Moreover, a mutation in MxiC that inhibits its interaction with MxiI (MxiC^F206S^), presents the same rescue effect. It was hypothesized that as this variant is secreted too early (before induction), it opens the way for effector secretion. Moreover, MxiI‐MxiC complexes could be physically implicated in the inhibition of effector secretion before host cell contact (Cherradi et al., 2013; El Hajjami et al., 2018).

Inactivation of *mxiC* or expression of MxiC^F206S^ in our “effector mutant” (Spa33^V192D^/Spa33^C^) also leads to effector secretion like in the wild‐type strain. These results suggest that Spa33 implicated as well in signal transmission/reception. We showed that Spa33 is able to interact directly with MxiC and MxiI, at least by its C‐terminal fragment. The interaction domain of Spa33 with MxiI and MxiC is probably different as the 3 proteins can form a complex in vitro. The interaction between MxiI with Spa33 was expected as Spa33‐MxiN‐MxiK complex acts as a “sorting platform” that determines the recognition, timing, and sorting of specific substrates exported in a defined order to form a functional T3SS (Hu et al., 2015). Moreover, most of the virulence proteins intended for secretion are produced by the bacteria during T3SS assembly and are believed to be predocked at the base of the *injectisome* until favorable conditions are available (Spaeth, Chen, & Valdivia, 2009). MxiC is also a secreted effector but its interaction with Spa33 could be more than a T3SS substrate‐secretion machinery interaction. Indeed, MxiC^F206S^ is able to rescue the phenotype of the effector mutant (Spa33^V192D^/Spa33^C^). Interestingly, the timing of MxiC^F206S^ secretion is restored to wild‐type level in the Spa33^V192D^/Spa33^C^ background that allows a wild‐type secretion profile. In the case of MxiC^F206S^, which maintained an interaction with Spa33, but failed to interact with MxiI (Cherradi et al., 2013), we observed a lack of secretion control before induction while secretion is normal following CR induction (Figure 5b,c). In the case of Spa33^V192D^/Spa33^C^, we can postulate that interaction between Spa33 and MxiC is stronger and leads to a defect of MxiC and effector secretion, in response to pore insertion.

## 5 CONCLUSIONS

In conclusion, we present evidences that *spa33* encodes multiple proteins that are required for T3SS function. For the first time, we show that Spa33 is involved in secretion hierarchy, regulating effector secretion upon host cell sensing. These results clearly indicate that Spa33 is involved in the timing of MxiC secretion. This study therefore provides comprehensive and critical insights into the complex regulation of T3SS and opens new avenues for future research endeavors.

## CONFLICT OF INTEREST

The authors declare no conflict of interest.

## AUTHOR CONTRIBUTIONS

MK, DL, and VD have done the experimental work and the acquisition of data. AB, MK, and PS have designed, analyzed and interpreted the data. DC and VI have done the MS experiments and their analysis. AB and MK have written the manuscript. DC, PS, DL, and VD have revised the manuscript.

## ETHICAL APPROVAL

None required.

## Data Availability

All data are provided in full in the results section of this paper.

## APPENDIX 1

**Table A1:** List of plasmids and strains used in this study

	Description	Source
Strains		
*S. flexneri* M90T	*S. flexneri* strain is a derivative of the wild‐type strain M90T (serotype 5) which is resistant to streptomycin	Allaoui, Sansonetti, and Parsot (1993)
*MxiC‐*	*mxiC* deficient mutant derived from *S. flexneri* M90T (serotype 5)	Botteaux et al. (2009)
MKA1	*spa33* mutant derived from *S. flexneri* M90T(serotype 5)	This study
MKA2	*mxiCspa33* double mutant derived from *S. flexneri* M90T (serotype 5)	This study
Plasmids		
pKD46	Plasmid used for expression of the λ Red system	Datsenko and Wanner (2000)
pMK1	pSU18Spa33 (His‐tag fused at COOH‐termini)	This study
pMK2	pSU18MxiCSpa33 (His‐tag fused at COOH‐termini)	This study
pMK3	pSU18Spa33^GTC^ (His‐tag fused at COOH‐termini)	This study
pMK4	pSU18Spa33^V192A^ (His‐tag fused at COOH‐termini)	This study
pMK5	pSU18Spa33^V192D^ (His‐tag fused at COOH‐termini)	This study
pMK6	pSU18MxiCSpa33^GTC^ (His‐tag fused at COOH‐termini)	This study
pMK7	pSU18MxiCSpa33^V192A^ (His‐tag fused at COOH‐termini)	This study
pMK8	pSU18MxiCSpa33^V192D^ (His‐tag fused at COOH‐termini)	This study
pMK9	pSU18MxiC^F206S^Spa33 (His‐tag fused at COOH‐termini)	This study
pMK10	pSU18MxiC^F206S^Spa33^V192D^ (His‐tag fused at COOH‐termini)	This study
pYC1	pSU18MxiCMxiH	Cherradi et al. (2013)
pAB30	pGEX4t1MxiC (GST‐tag fused at NH2‐termini)	Botteaux et al. (2009)
pAB117	pGEX4t1MxiC^F206S^ (GST‐tag fused at NH2‐termini)	Cherradi et al. (2013)
pYC178	pET30aMxiC (His‐tag fused at NH2‐termini)	Cherradi et al. (2013)
pSL24	pGEX4t1MxiI (GST‐tag fused at NH2‐termini)	Cherradi et al. (2013)
pMK11	pBADSpa33^C^ (His‐tag fused at COOH‐termini)	This study
pMK15	pGEX4T1Spa33 (GST‐tag fused at NH2‐termini)	This study
pMK16	pGEX4T1Spa33^V192D^ (GST‐tag fused at NH2‐termini)	This study
pMK17	pGEX4T1Spa33^C^ (GST‐tag fused at NH2‐termini)	This study
pMK18	pGEX4T1Spa33^C^His (GST‐tag fused at NH2‐termini and His‐tag fused at COOH‐termini)	
pMK19	pET30aSpa33his (His‐tag fused at both NH2‐ and COOH‐termini's)	This study
pMK20	pET30aSpa33 (His‐tag fused at NH2‐termini)	This study
pMK21	pET30aSpa33^GTC^his (His‐tag fused both at NH2‐ and COOH‐termini's)	This study
pMK22	pET30aSpa33^V192D^his (His‐tag fused both at NH2‐ and COOH‐termini's)	This study
pMK23	pET30aSpa33^V192A^his (His‐tag fused both at NH2‐ and COOH‐termini's)	This study
pMK24	pET30aSpa33^Slippage^ (mutated slippage site with silent mutation	This study
pMK25	pSU18MxiCSpa33^Slippage^ (mutated slippage site with silent mutation)	This study
pMK26	pBADSpa33 (His‐tag fused at COOH‐termini)	This study
pMK31	pBADSpa33^STOP^ (His‐tag fused at COOH‐termini)	This study
pMK32	pBADHisSpa33 (His‐tag fused at NH2‐termini)	This study

**Table A2:** List of primers used in this study. Introduced restriction sites are underlined

	Sequence 5′ – 3′	Restriction site
Primers for cloning		
Spa33BamHIs	5′ CCCGGATCCATGCTAAGAATTAAACATTTTGAC 3′	*Bam*HI
Spa33HindIIIas	5′ CCCAAGCTTTTAATGATGATGATGA TGATGCTCCTTTACCATCCAAGA 3′	*Hind*III
Spa33KpnIs	5′ CCCGGTACCGAACAGAGTGAAG AAGAATGCTAAGAATTAAACATTTTGAC 3′	*Kpn*I
Spa33PstIas	5′ CCCCTGCAGTTAATGATGATGATGAT GATGCTCCTTTACCATCCAAGA 3′	*Pst*I
Spa33^C^s	5′ CCCCCATGGGGGTGAATGATAATAATGAGGC 3′	*Nco*I
Spa33^C^as	5′ GTCGAAG CTT CTCCTTTACCATCC 3′	*Hind*III
Spa33BamHIs	5′ CCG‐GAT‐CCC‐TAA‐GAA‐TTA‐AAC‐ATT‐TTG‐AC 3′	*Bam*HI
Spa33XhoIas	5′ GGC‐TCG‐AGT‐TAC‐TCC‐TTT‐ACC‐ATC‐CAA‐GA 3′	*Xho*I
Spa33hisXhoIas	5′ CGC TCG AG TTA TGA TGA TGA TGA TGA TGA TGC TCC TTT ACC ATC ATC CAA GAA C 3′	*Xho*I
Primers for mutagenesis		
Spa33^GTC^s	5′ CAA‐GTT‐ATT‐ATT‐GGG‐GAT‐TAT‐ATT‐GTC‐AAC‐GAT‐AAT‐AAT‐GAG‐GCA‐AAA 3′	*Hinc*II
Spa33^GTC^as	5′ CTC‐ATT‐ATT‐ATC‐GTT‐GAC‐AAT‐ATA‐ATC‐CCC‐AAT‐AAT‐AAC‐TTG‐ATT‐ACA 3′	*Hinc*II
Spa33^V192A^s	5′ ATT‐GGG‐GAT‐TAT‐ATC‐GCG‐AAT‐GAT‐AAT‐AAT‐GAG‐GC 3′	*Nru*I
Spa33^V192A^as	5′ GCC‐TCA‐TTA‐TTA‐TCA‐TTC‐GCG‐ATA‐TAA‐TCC‐CCA‐AT 3′	*Nru*I
Spa33^V192D^s	5′ GGG GAT TAT ATC GAT AAT GAT AAT AAT GAG GC A AAA ATT AAT C 3′	*Cla*I
Spa33^V192D^as	5′ CTCATTATTATCATTATCGATATAATCCCCAATAATAACTTGATT 3′	*Cla*I
Spa33^M237A^s	GTG‐GAC‐TTT‐ATT‐CTT‐TTA‐GAA‐AAA‐AAC‐GCG‐ACA‐ATC‐AAT‐GAA‐CTA‐AAA‐ATG‐TAT‐G	
Spa33^M237A^as	CAT‐ACA‐TTT‐TTA‐GTT‐CAT‐TGA‐TTG‐TCG‐CGT‐TTT‐TTT‐CTA‐AAA‐GAA‐TAA‐AGT‐CCA‐C	
Spa33^slippage^s	5′ TCT‐TTT‐TTG‐AAG‐AAG‐AAA‐TAC‐GAG‐GTA 3′	
Spa33^slippage^as	5′ GTA‐TTT‐CTT‐CTT‐CAA‐AAA‐AGA‐TAA‐TGT‐ATC 3′	
Spa33^STOP^s	5′ GAT‐TAT‐ATT‐GTG‐TAA‐GAT‐AAT‐AAT‐GAG‐GCA AAA ‐ATT ‐AAT ‐CTG 3′	
Spa33^STOP^as	5′ CTC‐ATT‐ATT‐ATC‐TTA‐CAC‐AAT‐ATA‐ATC‐CCC‐AAT‐AAT‐AAC‐TTG 3′	
Primers for knockout		
Spa32full s	5′ ATGGCATTAGATAATATAAACCTA 3′	
Spa32Kmas	5′ TTCCTCCTAGTTAGTCACTTAGCATTCTTCTTCACTCTGTTCATT 3′	
Kmspa32s	5′ AGTGAAGAAGAATGCTAAGGTGACTAACTAGGAGGAATAAATGGC 3′	
Kmspa24as	5′ GGACATGTCACTCAGCATCTAAAACAATTCATCCAGTAAAATATA 3′	
Kmspa24s	5′ CTGGATGAATTGTTTTAGTCTCATGCTGAGTGACATGTCCCTCATCGCT 3′	
Spa24fullas	5′ CTAAGCAGGAATATTGATATAT 3′	

**Table A3:** Peptides recovered from trypsin digestion and MS analysis of Spa33^C^ with confidence of more than 90%

Time	Prec MW	Prec *m*/*z*	Prec *z*	Prot *N*	Best Sequence	Modifications	Conf	Theor MW	*z*	Position in Spa33
78,085	1,169,542	5,857,784	2	4	MYVENELFK	Met‐>Glu@1	99	1,169,56	2	244–251
83,997	1,164,588	5,833,014	2	4	GIEISSWMVK	Oxidation(M)@8	99	1,164,585	2	283–292
109,075	2,001,975	6,683,324	3	4	MYVENELFKFPDDIVK	Oxidation(M)@1	99	2,001,976	3	244–259
110,483	1,983,967	6,623,297	3	4	MYVENELFKFPDDIVK	Oxidation(M)@1, Dehydrated(Y)@2	99	1,983,965	3	244–259
73,365	1,187,554	5,947,843	2	4	MYVENELFK	Oxidation(M)@1	98	1,187,553	2	244–251
